# Lipid-Rich Plaque Beneath Dense Calcium: Imaging-Guided Lithotripsy in Calcified Double-Vessel Disease

**DOI:** 10.1016/j.jaccas.2026.110264

**Published:** 2026-10-07

**Authors:** Syed Nazar Raza, Md Rehan Akhtar, Manav Jyala, Syed Abeera Andrabi

**Affiliations:** a Department of Allied and Healthcare Sciences, Jamia Hamdard, New Delhi, India; b Innovative Therapeutic Pvt. Ltd., Chennai, Tamil Nadu, India

**Keywords:** coronary calcification, intravascular lithotripsy, intravascular ultrasound, lipid core plaque, near-infrared spectroscopy, stent expansion

## Abstract

**Background:**

Grayscale intravascular ultrasound grades coronary calcium but cannot detect lipid sequestered behind a calcific plate because both attenuate the ultrasound signal.

**Case Summary:**

A 56-year-old man with palpitations and exertional chest discomfort had double-vessel disease: a critical proximal left anterior descending (LAD) stenosis and a severe mid-to-distal right coronary artery (RCA) lesion. Coregistered near-infrared spectroscopy and intravascular ultrasound showed near-circumferential calcium in both vessels, with a large lipid core hidden beneath the LAD calcific shell (maximum lipid core burden index over 4 mm: 788) and a lipid-poor RCA focus (maximum lipid core burden index over 4 mm: 249). Lithotripsy and drug-eluting stents were used in both vessels. The lipid-rich LAD expanded well; the lipid-poor RCA focus did not.

**Take-Home Message:**

Plaque chemistry, not calcium geometry alone, may explain why some calcified segments resist lithotripsy.

## Visual Summary

**Figure:**
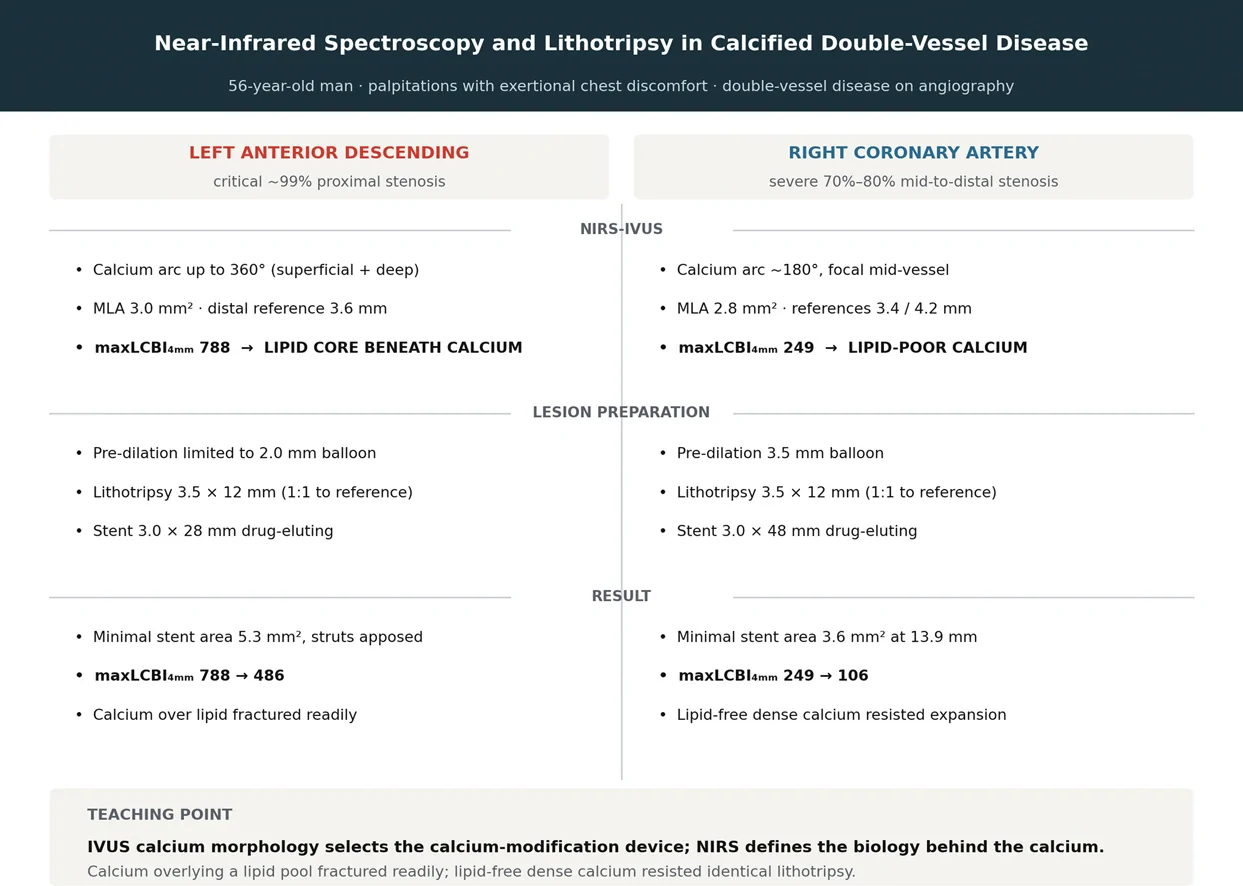

Heavily calcified lesions remain among the least forgiving substrates in percutaneous coronary intervention: Calcium obstructs device delivery and symmetrical stent expansion, predisposing to malapposition, restenosis, and stent thrombosis.^1^ Fluoroscopy underestimates calcium burden,^2^ and although grayscale intravascular ultrasound (IVUS) measures calcium arc and length well enough to forecast underexpansion,^3^ it cannot distinguish dense fibrocalcific tissue from lipid behind it because both attenuate the signal. Near-infrared spectroscopy (NIRS) interrogates plaque chemically and, coregistered with IVUS on one catheter, maps lipid core irrespective of an overlying calcific plate.^4^^,^^5^ We report a patient with calcified double-vessel disease in whom NIRS-IVUS disclosed lipid concealed beneath calcium, and how that finding shaped device selection and stent coverage.

## History of Presentation

A 56-year-old man was referred for palpitations of several weeks' duration with exertional chest discomfort that remitted predictably on rest. He was hemodynamically stable, with a pulse 80 beats/min and regular and blood pressure 130/80 mm Hg. Heart sounds were normal without murmur or gallop, the jugular venous pressure was not elevated, and respiratory, abdominal, and neurological examinations were unremarkable.

## Past Medical History

His cardiovascular risk profile comprised hypertension, diabetes, smoking, dyslipidemia, and previous myocardial infarction.

## Differential Diagnosis

Effort-limited discomfort remitting on rest placed obstructive coronary disease at the head of the differential. Palpitations raised the alternative of a primary atrial or ventricular arrhythmia, incidental or ischemia provoked, addressed by 12-lead findings. Musculoskeletal chest wall pain, gastroesophageal reflux, and anxiety-related discomfort were improbable given the reproducible effort threshold, and aortic stenosis and hypertrophic cardiomyopathy were excluded on examination and echocardiography, so invasive evaluation was preferred to further noninvasive testing.

## Investigations

### Diagnostic coronary angiography

Angiography via the right radial artery showed double-vessel disease: a critical (∼99%) proximal left anterior descending artery (LAD) stenosis with minor diagonal disease, a 50% to 60% distal left circumflex stenosis with minor obtuse marginal disease, and a severe 70% to 80% mid to distal right coronary artery (RCA) stenosis with minor branch disease (Figure 1, Figure 2, Figure 3). Both target lesions were densely calcified on fluoroscopy, so coregistered NIRS-IVUS (Makoto Intravascular Imaging System, Infraredx) was used to guide intervention.

**Figure 1:**
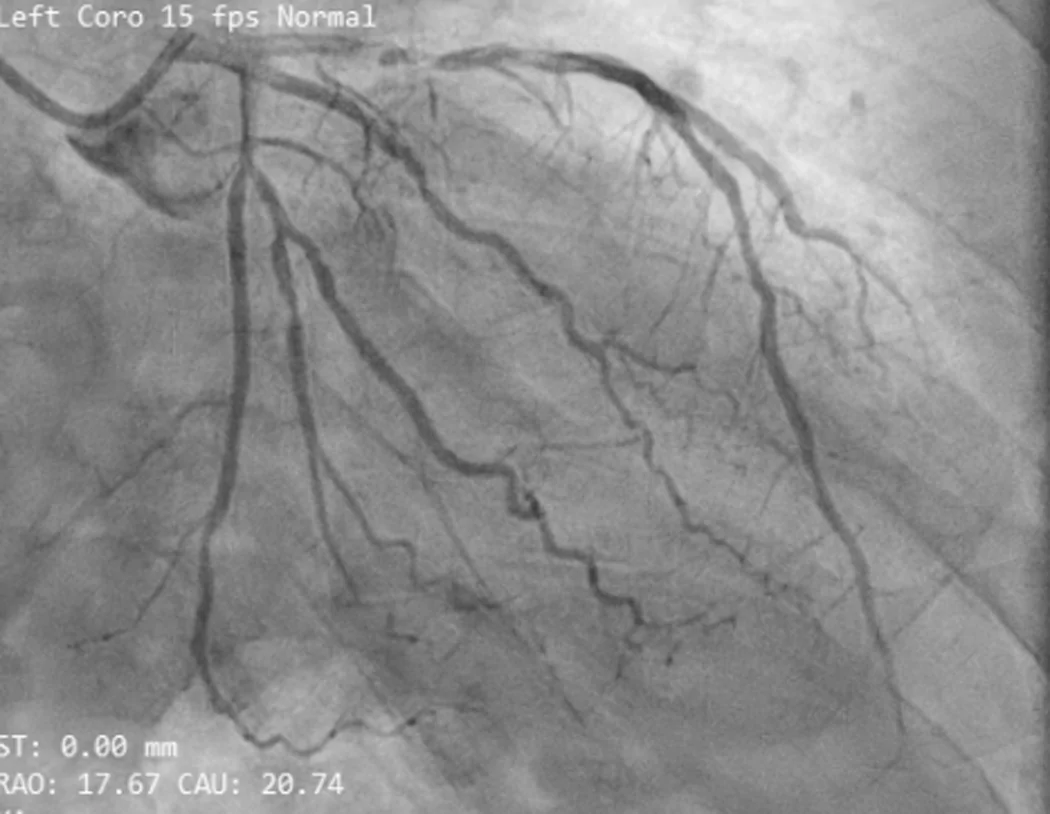
Baseline Left Coronary Angiogram, Right Anterior Oblique Caudal Projection (RAO 18°, Caudal 21°) The left main stem is widely patent. This projection opens the circumflex territory and shows moderate narrowing (50%-60%) of the distal left circumflex artery, with only minor irregularity of the obtuse marginal branches. Neither lesion was judged to require treatment at this sitting, and the view was obtained to separate the circumflex from the overlapping left anterior descending artery before intervention was planned. RAO = right anterior oblique.

**Figure 2:**
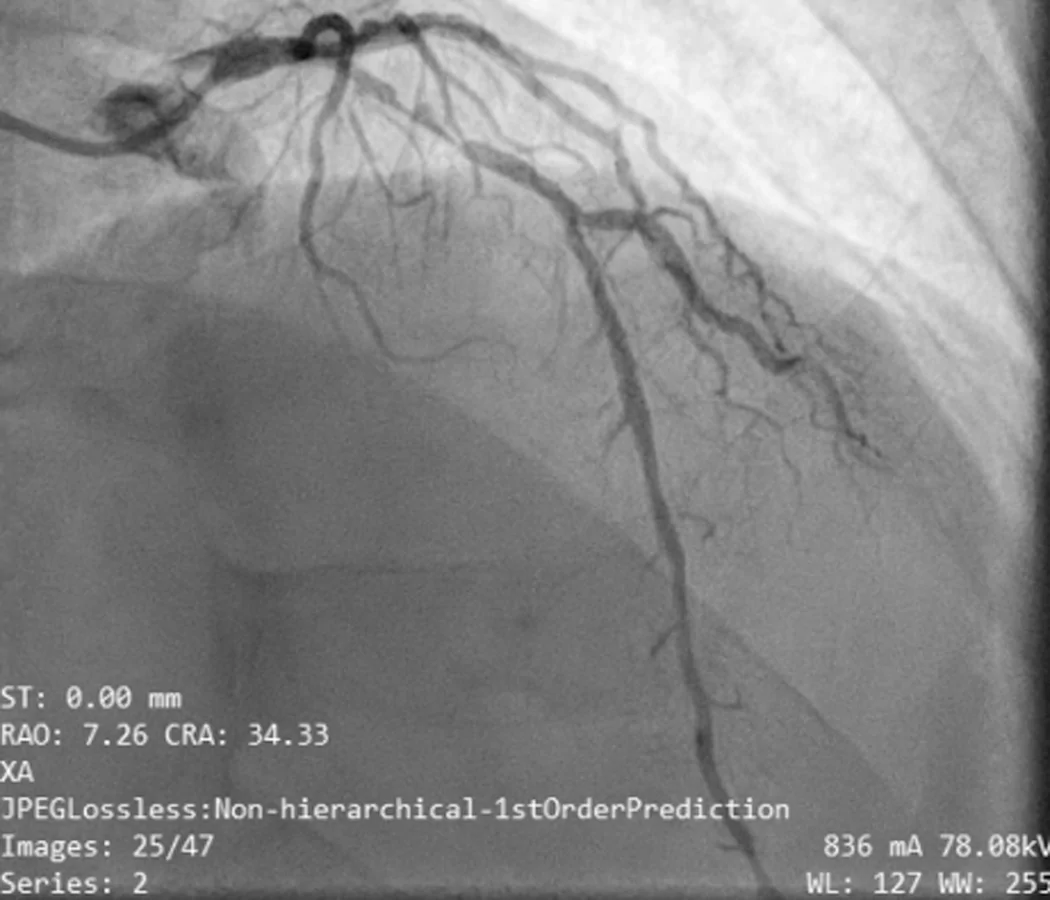
Baseline Left Coronary Angiogram, Right Anterior Oblique Cranial Projection (RAO 7°, Cranial 34°) Foreshortening of the proximal segment is minimized in this projection, which displays the critical (∼99%) proximal left anterior descending artery (LAD) narrowing that was the principal culprit lesion. Antegrade filling beyond the stenosis is delayed, and the vessel caliber distal to it is poorly opacified. The diagonal branch carries only minor plaque. Mural radiodensity along the proximal LAD on unsubtracted fluoroscopy corresponded to the calcification later quantified by intravascular ultrasound. RAO = right anterior oblique.

**Figure 3:**
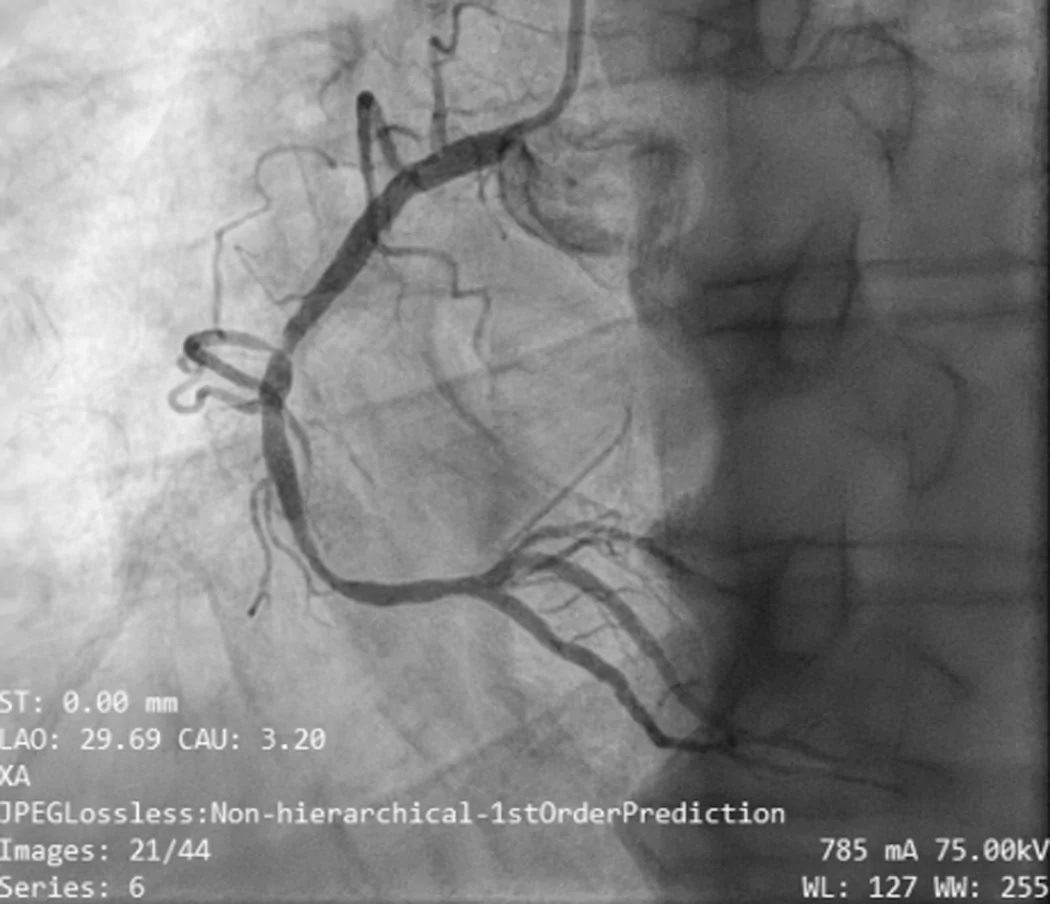
Baseline Right Coronary Angiogram, Left Anterior Oblique Projection (LAO 30°, Caudal 3°) The right coronary artery is dominant. A severe narrowing of 70% to 80% involves the mid to distal vessel proximal to the crux, and the posterior descending and posterolateral branches carry only minor disease. Heavy mural calcification was apparent in this segment on fluoroscopy, which prompted intravascular imaging rather than direct stenting. LAO = left anterior oblique.

### Coregistered NIRS-IVUS assessment

In the LAD, the distal reference lumen diameter was 3.6 mm, the minimal lumen area was 3.0 mm^2^, and the maximum lipid core burden index over 4 mm (maxLCBI4mm) was 788. Grayscale imaging showed a 160° calcific arc at the culprit, focal calcium reaching 170° proximally, and a complete 360° shell distally at a side-branch origin. Decisively, the chemogram identified a lipid core beneath that calcified segment, which grayscale imaging did not reveal (Figures 4 and 5).

**Figure 4:**
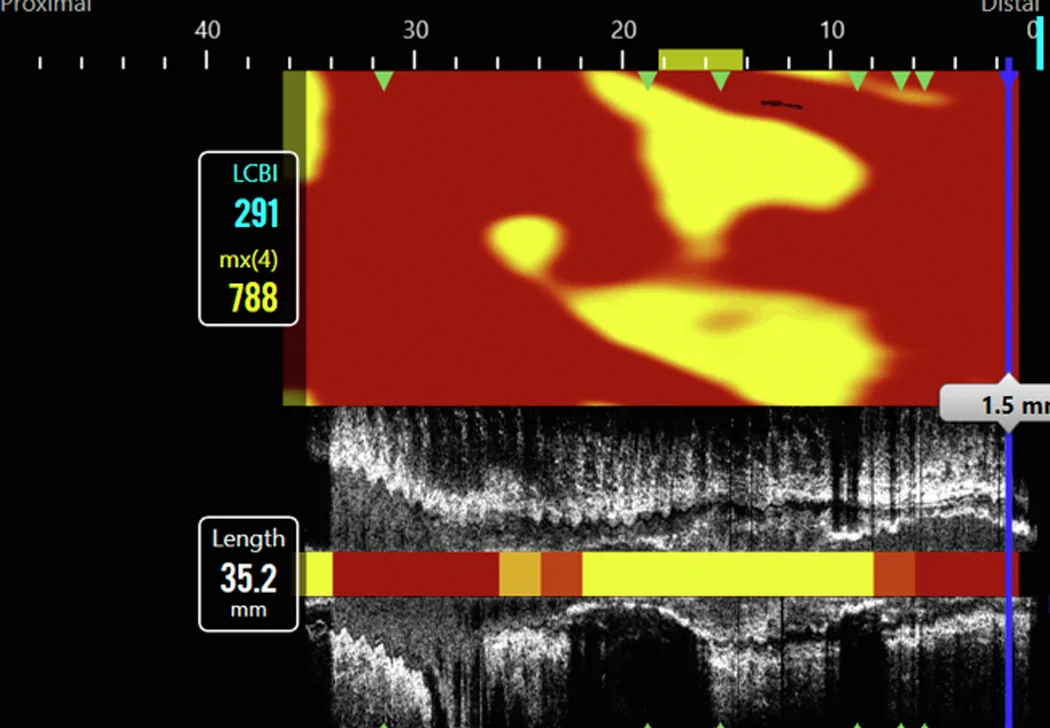
Preintervention Near-Infrared Spectroscopy and Intravascular Ultrasound of the LAD Chemogram (upper panel), block chemogram (central band), and longitudinal grayscale ultrasound over a 35.2-mm pullback. Broad confluent yellow signal marks an extensive lipid core, giving a whole-pullback lipid core burden index of 291 and a maxLCBI4mm of 788. The lipid maps to the segment where grayscale ultrasound shows a bright superficial calcific interface with deep acoustic shadowing, so the underlying pool would have been invisible on ultrasound alone. The distinction is the pivot of this case: It excluded ablative modification and set the length of stent required to bury the pool. LAD = left anterior descending artery; maxLCBI4mm = maximum lipid core burden index over 4 mm.

**Figure 5:**
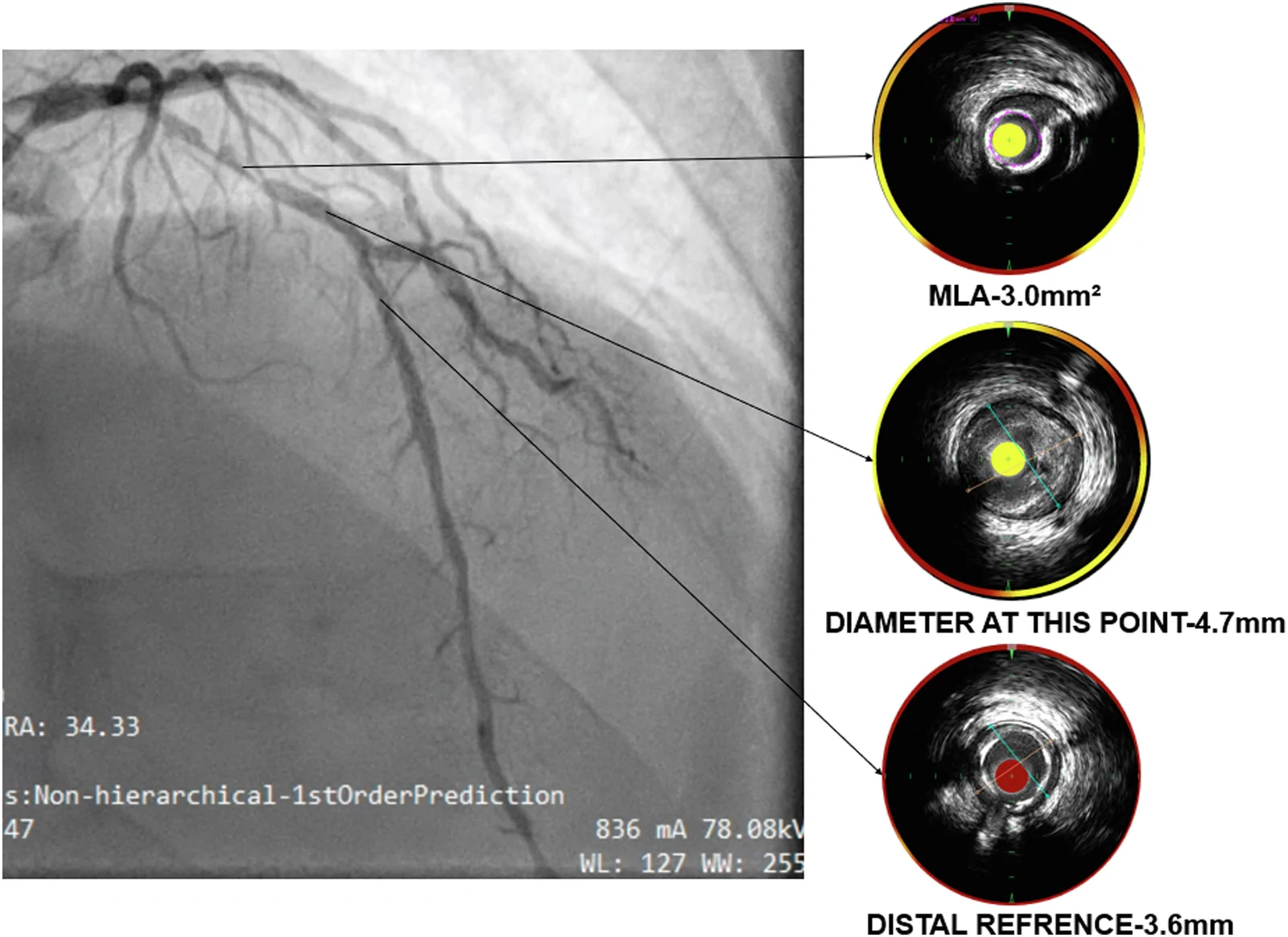
Coregistered Near-Infrared Spectroscopy and Intravascular Ultrasound of the LAD Angiogram (left) with ultrasound cross-sections taken at the 3 levels indicated by the arrows (right). Top: minimal lumen area (MLA) 3.0 mm^2^ at the culprit, where a dense superficial calcific arc occupies roughly 160° to 170° of the circumference and casts a shadow that obscures the plaque behind it. Middle: lumen diameter 4.7 mm at the proximal reference. Bottom: distal reference diameter 3.6 mm, the value used to size the lithotripsy balloon. The colored ring encircling each section is the chemogram coregistered to that level, and yellow arcs signify lipid lying deep to the calcific plate. LAD = left anterior descending artery.

The RCA showed a distal reference of 3.2 mm, a proximal reference of 4.2 mm, a minimal lumen area of 2.8 mm^2^, and a much lower maxLCBI4mm of 249, with mixed fibrolipidic plaque and an approximately 180° calcific arc at a discrete, tightly stenotic midvessel segment (Figures 6 and 7). A maxLCBI4mm approaching or exceeding 400 to 500 has been linked to periprocedural myocardial infarction, no reflow, and distal embolization^6^^,^^7^ (Figure 8, Table 1).

**Figure 6:**
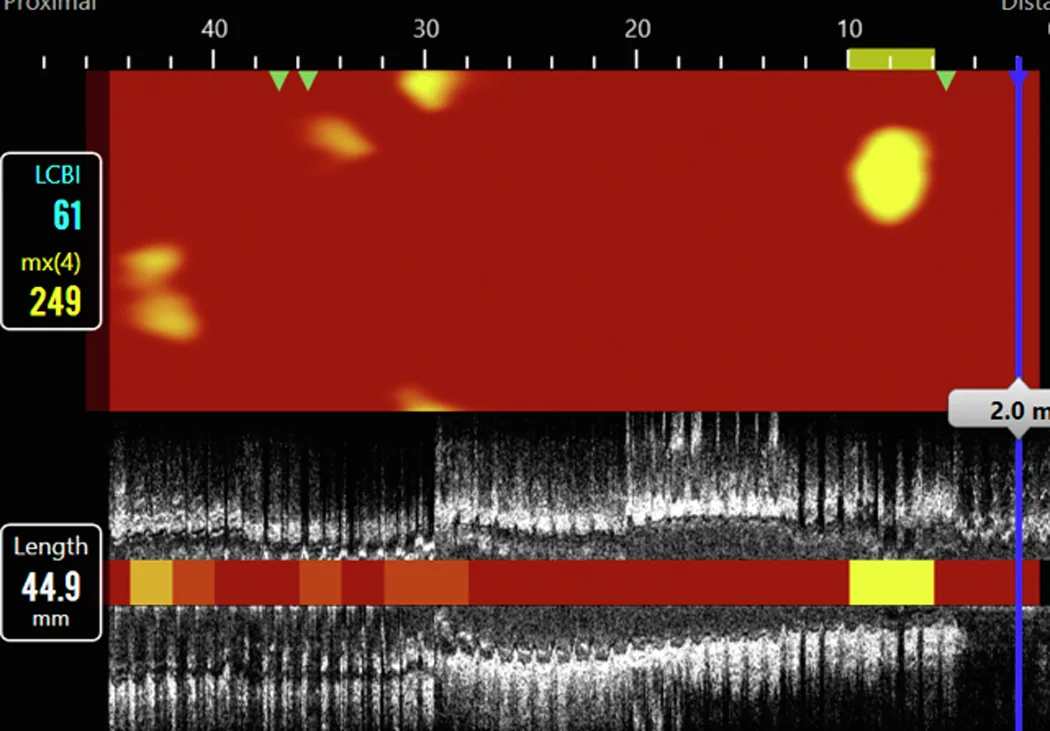
Preintervention Near-Infrared Spectroscopy and Intravascular Ultrasound of the RCA Corresponding display over a 44.9-mm pullback. The chemogram (upper panel) is almost uniformly red apart from a small discrete yellow focus distally and scattered orange proximally, yielding a lipid core burden index of 61 and a maxLCBI4mm of 249. Longitudinal ultrasound nevertheless shows repetitive bright reflectors with dense shadowing along much of the segment, indicating that the plaque is calcified but lipid-poor. Read against Figure 4, the pair illustrates 2 lesions that look comparably calcified on ultrasound yet differ fundamentally in composition. RCA = right coronary artery; maxLCBI4mm = maximum lipid core burden index over 4 mm.

**Figure 7:**
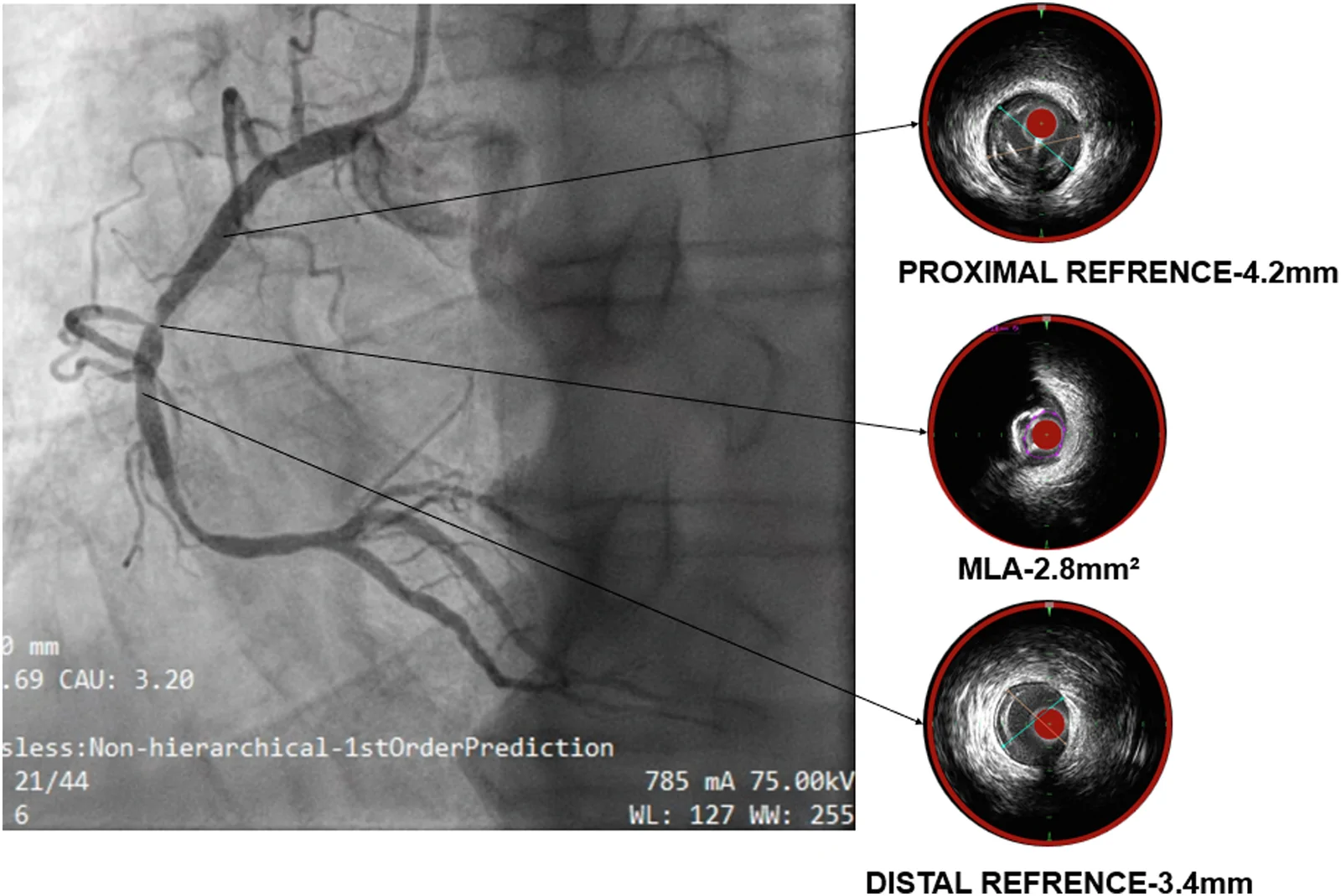
Coregistered Near-Infrared Spectroscopy and Intravascular Ultrasound of the RCA Angiogram (left) with the corresponding cross-sections indicated by arrows (right). Top: proximal reference diameter 4.2 mm. Middle: minimal lumen area (MLA) 2.8 mm^2^ at the midvessel focus, where calcium subtends approximately 180°. Bottom: distal reference diameter 3.4 mm. The chemogram rings are red throughout, confirming that this densely calcified segment is lipid poor and providing the internal control for the LAD findings in Figure 5. LAD = left anterior descending artery; RCA = right coronary artery.

**Figure 8:**
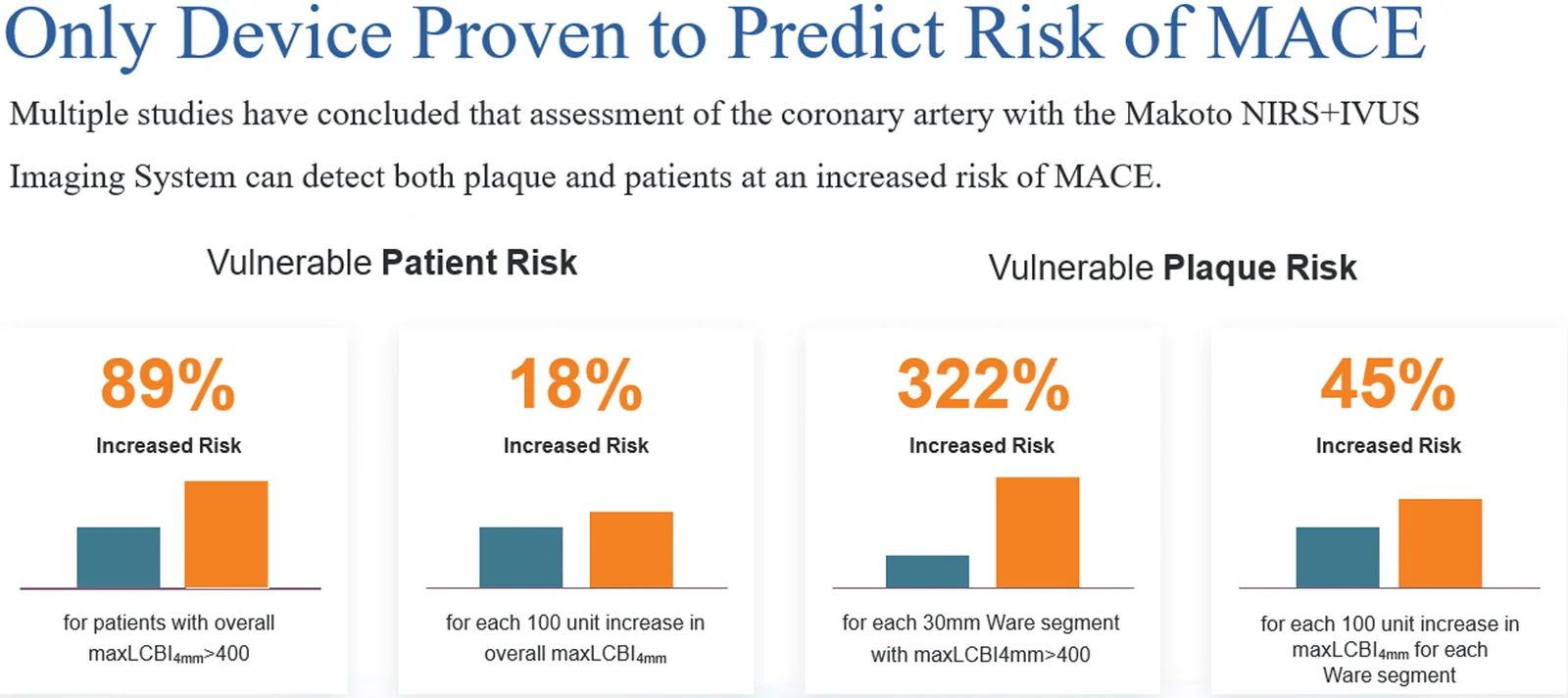
Reported Relationship Between Lipid Core Burden and Subsequent Adverse Cardiovascular Events Summary graphic of published outcome data obtained with near-infrared spectroscopy and intravascular ultrasound, expressing patient-level and plaque-level excess risk of major adverse cardiovascular events for a maxLCBI4mm of >400 and for each 100-unit rise in maxLCBI4mm. The graphic frames the prognostic weight of the maxLCBI4mm value of 788 recorded in this patient's LAD and the comparatively modest value of 249 in the RCA. IVUS = intravascular ultrasound; LAD = left anterior descending artery; MACE = major adverse cardiovascular events; maxLCBI4mm = maximum lipid core burden index over 4 mm; NIRS = near-infrared spectroscopy.

**Table 1:** Imaging and Procedural Characteristics of the 2 Treated Vessels

	Left Anterior Descending Artery	Right Coronary Artery
Angiographic diameter stenosis	∼99% (proximal)	70%-80% (mid to distal)
Reference lumen diameter	3.6 mm (distal)	4.2 mm (proximal); 3.2 mm (distal)
Minimal lumen area	3.0 mm^2^	2.8 mm^2^
Maximum calcium arc	160°-170° at culprit; 360° distally	∼180° at midvessel focus
Plaque phenotype on grayscale	Fibrocalcific with attenuation	Mixed fibrolipidic
maxLCBI4mm before intervention	788	249
Predilation balloon	2.0 × 10 mm	3.5 × 10 mm
Lithotripsy balloon	3.5 × 12 mm	3.5 × 12 mm
Drug-eluting stent	3.0 × 28 mm	3.0 × 48 mm
Minimal stent area	5.3 mm^2^	3.6 mm^2^ at 13.9 mm; 4.8-8.8 mm^2^ elsewhere
maxLCBI4mm after intervention	486	106

Reference diameters and lumen areas are intravascular ultrasound measurements. The nominal cross-sectional area of a 3.0-mm stent is 7.1 mm^2^, so the achieved minimal stent areas correspond to approximately 75% (LAD) and 51% (RCA focus) of nominal.

maxLCBI4mm = maximum lipid core burden index over 4 mm (ie, the 4-mm segment of the interrogated vessel carrying the greatest concentration of lipid core plaque on the chemogram).

## Management

### Choice of calcium-modification device

Three imaging features drove device selection. First, calcium was circumferential rather than eccentric—160° to 170° at the LAD culprit with a 360° shell distally, and approximately 180° in the RCA—a morphology that meets published IVUS criteria for anticipated underexpansion^3^ and one that a cutting or scoring balloon, acting on discrete points of an eccentric plate, modifies inconsistently. Second, the LAD calcium overlaid a large lipid pool. Rotational and orbital atherectomy remove tissue and liberate particulate debris, and applying either above a plaque with a maxLCBI4mm of 788—far beyond the threshold at which periprocedural infarction becomes frequent^6^^,^^7^—was judged to carry an unfavorable embolic profile. Intravascular lithotripsy instead fractures superficial and deep calcium in situ by acoustic pressure waves at low balloon pressure, without debulking, and it was therefore chosen because of the chemogram rather than in spite of it. Third, both lesions crossed readily with a workhorse wire and a small balloon, so atherectomy was not required for passage.

### Procedure

Percutaneous coronary intervention was performed via the right radial artery with a 7-F guiding catheter. The LAD was wired conventionally, predilated with a 2.0 × 10 mm balloon, and was treated with a 3.5 × 12 mm lithotripsy balloon delivering 10 pulses per cycle and 4 atm (Figures 9A to 9D). A 3.0 × 28 mm drug-eluting stent was deployed from the proximal to mid LAD, the length chosen to bury the whole chemogram-defined lipid core rather than land an edge upon it, since lipid left uncovered at a stent margin carries adverse late implications.^8^ Poststent imaging showed a minimal stent area of 5.3 mm^2^ with well-apposed struts, no edge dissection, and a fall in maxLCBI4mm to 486 (Figure 10).

**Figure 9:**
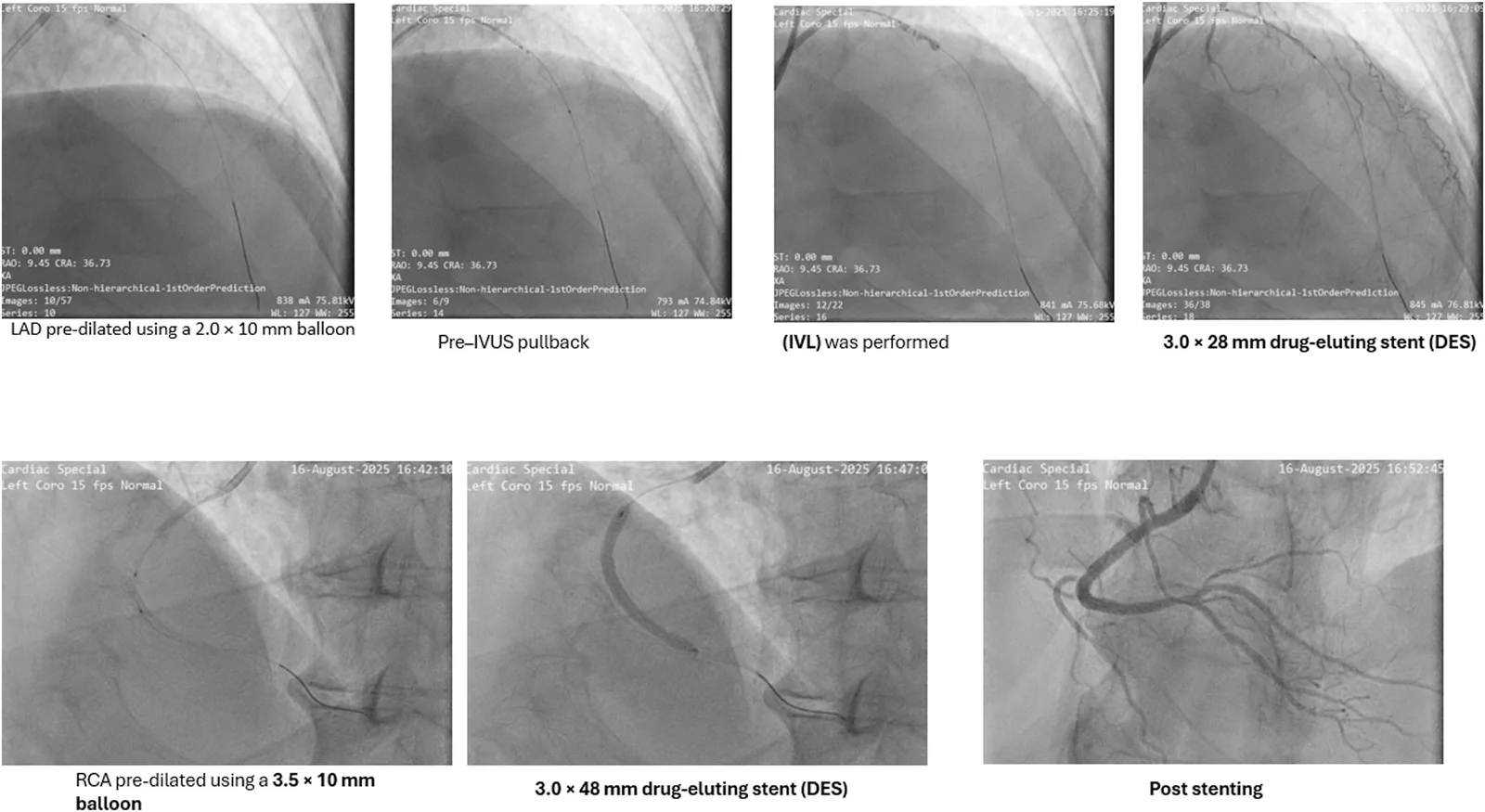
Procedural Sequence in Both Vessels (Top, left to right) Predilation of the LAD with a 2.0 × 10 mm balloon. Positioning of the imaging catheter for the LAD pullback. Intravascular lithotripsy (IVL) of the LAD with a 3.5 × 12 mm balloon delivering 10 pulses per cycle at 4 atm. Deployment of a 3.0 × 28 mm drug-eluting stent from the proximal to the mid LAD; the length was chosen so that the chemogram-defined lipid core lay entirely within the stented segment rather than at an edge. (Bottom, left to right) Predilation of the RCA with a 3.5 × 10 mm balloon. Deployment of a 3.0 × 48 mm drug-eluting stent across the mid to distal RCA. Final angiogram after treatment of both vessels, showing brisk antegrade flow with no residual stenosis, edge dissection, perforation or slow flow. LAD = left anterior descending artery; RCA = right coronary artery.

**Figure 10:**
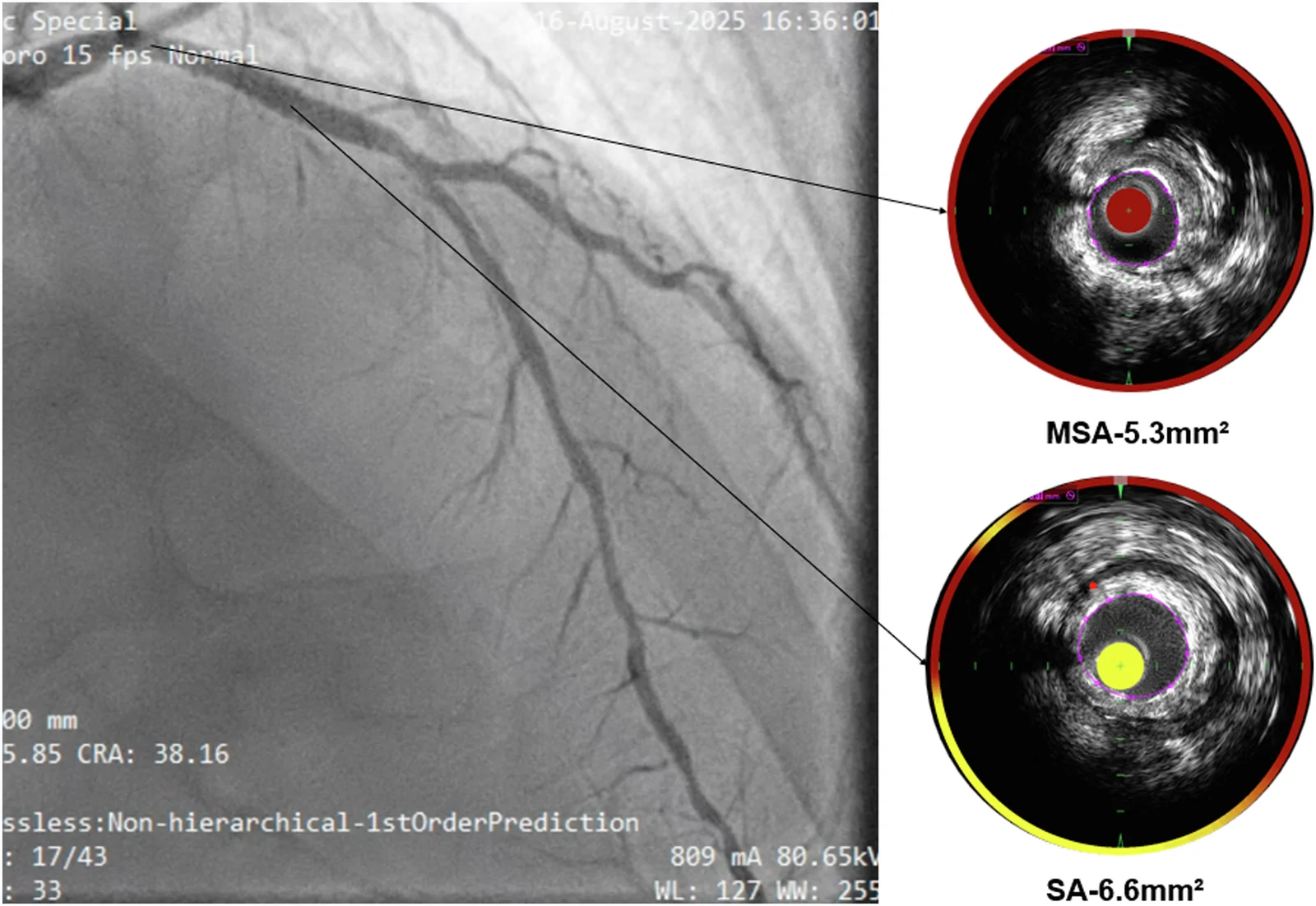
Poststent Assessment of the LAD (Left) Angiogram after stenting, with intravascular ultrasound cross-sections at the 2 levels indicated by arrows (Right). Top: minimal stent area (MSA) 5.3 mm^2^ at the most constrained point within the stent, with struts symmetrically apposed to the vessel wall and no edge dissection. Bottom: stent area (SA) 6.6 mm^2^ in an adjacent segment, where a yellow arc persists on the coregistered chemogram. The residual signal, with the maxLCBI4mm value falling from 788 to 486, indicates lipid now sealed behind apposed struts rather than lipid removed; this was the basis for intensified lipid-lowering therapy. LAD = left anterior descending artery; maxLCBI4mm = maximum lipid core burden index over 4 mm.

The RCA sequence was analogous: predilation with a 3.5 × 10 mm balloon, lithotripsy with a 3.5 × 12 mm balloon, and a 3.0 × 48 mm drug-eluting stent across the mid-to-distal segment. Minimal stent area was 3.6 mm^2^ at the most resistant, densely calcified subsegment and 13.9 mm along the pullback, rising to 4.8 to 8.8 mm^2^ elsewhere, with maxLCBI4mm falling to 106 (Figures 9E to 9G and 11). That focal underexpansion corresponded precisely to a segment where the chemogram showed almost no lipid.

**Figure 11:**
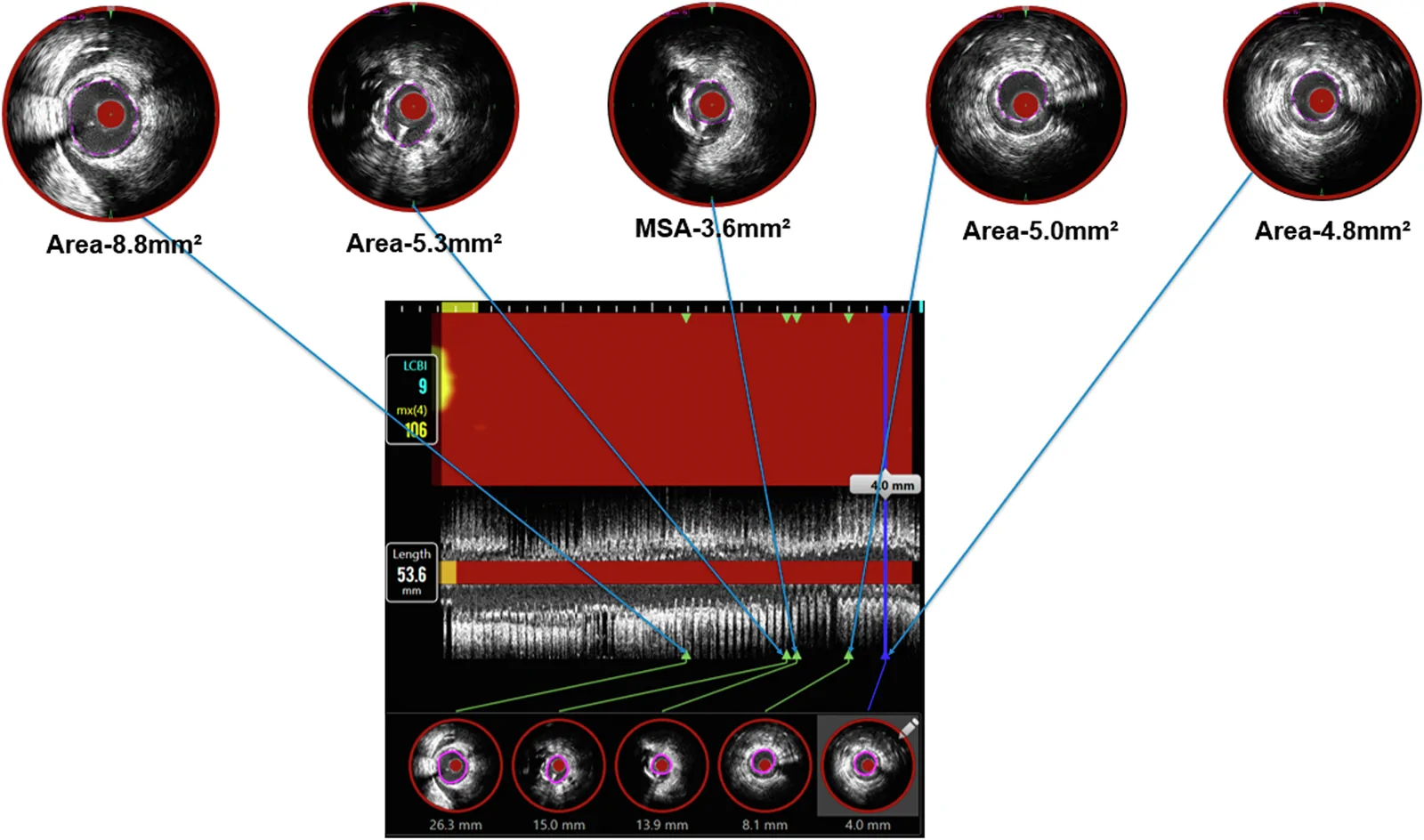
Poststent Assessment of the RCA Chemogram and longitudinal ultrasound over a 53.6-mm pullback (Center) with cross-sections at the positions indicated by the arrows (Top). Stent area measures 8.8 mm^2^ at 26.3 mm and 5.3 mm^2^ at 15.0 mm, falls to a minimal stent area (MSA) of 3.6 mm^2^ at 13.9 mm, and recovers to 5.0 mm^2^ at 8.1 mm and 4.8 mm^2^ at 4.0 mm. The point of greatest underexpansion coincides with the densely calcified subsegment at which the chemogram shows virtually no lipid. The lipid core burden index fell to 9 and the maxLCBI4mm to 106. Taken with Figure 10, the comparison shows lipid-rich and lipid-poor calcium behaving differently after identical lithotripsy. LAD = left anterior descending artery; maxLCBI4mm = maximum lipid core burden index over 4 mm; RCA = right coronary artery.

**Visual Summary:**
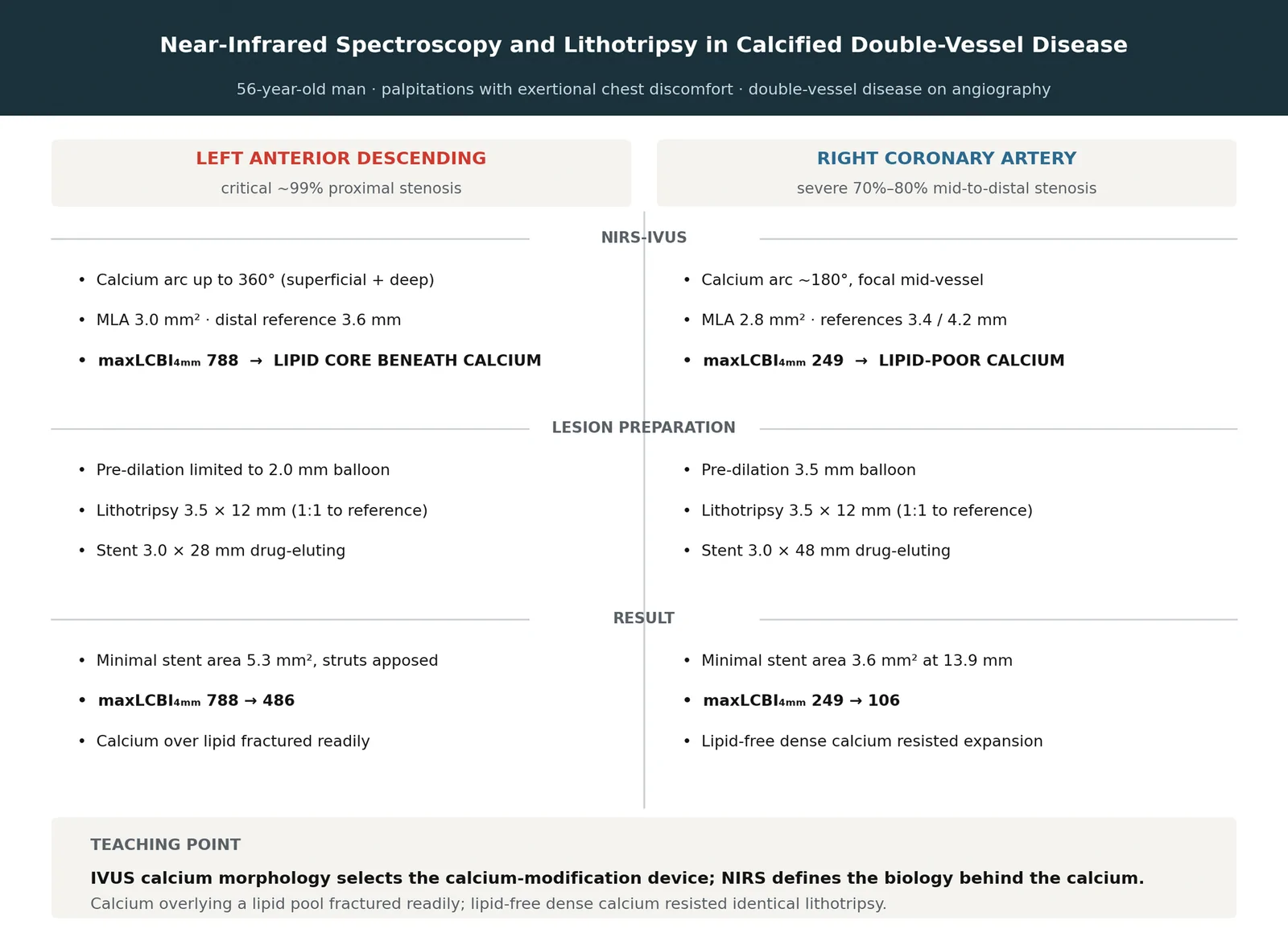
Imaging-Guided Lithotripsy in Calcified Double-Vessel Disease Timeline from presentation to procedural outcome, with vessel-level imaging and device data and the resulting teaching points. The lipid-rich left anterior descending artery calcification expanded after lithotripsy, whereas the lipid-poor focal right coronary artery calcification did not. IVUS = intravascular ultrasound; maxLCBI4mm = maximum lipid core burden index over 4 mm; MLA = minimal lumen area; NIRS = near-infrared spectroscopy.

### Rationale for balloon and stent sizing

Lithotripsy balloons are sized 1:1 to the reference vessel and inflated to low pressure; oversizing risks dissecting a rigid conduit, undersizing limits wall apposition and hence calcium fracture. With reference diameters of 3.6 mm in the LAD and 4.2 mm proximally and 3.2 mm distally in the RCA, a 3.5-mm device was the closest available approximation to 1:1 in both vessels. Stent diameter was chosen conservatively at 3.0 mm: The RCA stent had to span 48 mm into a tapering distal segment, and in the LAD the operator sized to the smaller distal reference to limit edge dissection or perforation through freshly fractured calcium. In retrospect, this was conservative: Minimal stent areas of 5.3 mm^2^ and 3.6 mm^2^ represent approximately 75% and 51% of the nominal area of a 3.0-mm device, so postdilation with a noncompliant balloon, or a 3.5-mm stent in the LAD, would have conformed more closely to contemporary expansion targets.

## Follow-Up

The procedure was completed without dissection, perforation, slow flow, or angiographic evidence of distal embolization, and the patient remained hemodynamically stable throughout. Given the residual lipid signal within the stented LAD, high-intensity lipid-lowering therapy was continued alongside dual antiplatelet therapy, and risk-factor modification was reinforced.

## Discussion

NIRS did not determine whether the calcium needed modification: that followed from the IVUS calcium arc and length, and lithotripsy would probably have been chosen on grayscale imaging alone. What the chemogram changed was how the calcium was modified and what was done around it. It argued against ablative modification of the LAD for the embolic reasons above; it defined the longitudinal limits of the lipid pool and hence the stent length needed to cover it^8^; it prompted staged low-pressure preparation with readiness for slow flow rather than empirical high-pressure dilation; and it supplied a compositional endpoint unavailable to ultrasound—the fall in maxLCBI4mm to 486 and 106 (Figures 10 and 11)—documenting that the lipid core had been sealed beneath apposed struts. Residual lipid signal within the stented LAD justified intensified lipid-lowering therapy and closer surveillance.

A second and less anticipated contribution was mechanical. A compliant lipid pool behind a calcific plate removes the rigid backing against which acoustic energy must act, so calcium overlying lipid should fracture more readily than calcium seated on fibrous or calcific tissue. This case contains its own internal comparison: The LAD, with a maxLCBI4mm of 788 and arcs reaching 360°, expanded satisfactorily, whereas the RCA remained underexpanded at exactly the point where the chemogram showed almost no lipid. Lipid behind calcium may therefore modulate the response to lithotripsy, and NIRS may prospectively identify lipid-poor, hard-backed calcium requiring additional preparation. Derived from one patient, this is a hypothesis rather than a demonstration.

Randomised data confirm that lithotripsy modifies severe calcium safely,^9^ while ECLIPSE indicates that imaging-guided balloon-based preparation suffices for most crossable calcified lesions^10^ — the value of imaging lying less in reflexive device escalation than in matching preparation to substrate.

### Limitations

This is a single uncontrolled observation without repeat imaging or long-term follow-up, and stent expansion fell short of contemporary targets, particularly at the calcified RCA focus, so periprocedural myocardial infarction cannot be excluded angiographically. Finally, the inference that lipid behind calcium facilitated lithotripsy rests on a 2-vessel comparison within a single patient and does not establish mechanism.

## Conclusions

Coregistered NIRS-IVUS altered the interpretation of 2 densely calcified lesions by revealing a lipid core hidden beneath a calcific shell. That finding steered preparation away from debulking toward lithotripsy and defined stent coverage. The divergent response of lipid-rich and lipid-poor calcified segments to identical lithotripsy suggests that plaque chemistry, not calcium geometry alone, may help predict which calcified lesions expand.

**Take-Home Messages**

• When a lesion appears densely calcified on angiography or grayscale intravascular ultrasound, near-infrared spectroscopy can reveal a hidden lipid core and reshape lesion preparation.
• A high maximum lipid core burden index over 4 mm argues against debulking, defines the stent length needed to cover the lipid pool, and warrants heightened periprocedural vigilance.
• Focal, lipid-poor calcium can resist lithotripsy even when adjacent lipid-rich segments expand well, and should prompt escalation rather than acceptance of the result.

## Funding Support and Author Disclosures

The authors have reported that they have no relationships relevant to the contents of this paper to disclose.
